# Dynamic serum biomarkers to predict the efficacy of PD-1 in patients with nasopharyngeal carcinoma

**DOI:** 10.1186/s12935-021-02217-y

**Published:** 2021-09-28

**Authors:** Ao Zhang, Guanqing Zhong, Luocan Wang, Rongzeng Cai, Runkun Han, Caixia Xu, Shulin Chen, Peng Sun, Hao Chen

**Affiliations:** 1 Department of Clinical Laboratory, State Key Laboratory of Oncology in South China, Collaborative Innovation Center for Cancer Medicine, Guangdong Key Laboratory of Nasopharyngeal Carcinoma Diagnosis and Therapy, Sun Yat-Sen University Cancer Center, Guangzhou, 510060 People’s Republic of China; 2 Research Center for Translational Medicine, the First Affiliated Hospital, Sun Yat-Sen University, 58 Zhongshan Road 2, Guangzhou, Guangdong 510080 P.R. China; 3 Department of Medical Oncology, State Key Laboratory of Oncology in South China, Collaborative Innovation Center for Cancer Medicine, Guangdong Key Laboratory of Nasopharyngeal Carcinoma Diagnosis and Therapy, Sun Yat-Sen University Cancer Center, Guangzhou, 510060 People’s Republic of China

**Keywords:** Biomarker, PD-1, Nasopharyngeal carcinoma, ICB, Dynamic monitor

## Abstract

**Background:**

There is a lack of effective treatments for recurrent or metastatic nasopharyngeal carcinoma (RM-NPC). Furthermore, the response rate of NPC patients to programmed death 1 (PD-1) inhibitors is approximately 20% to 30%. Thus, we aimed to explore reliable and minimally invasive prognostic indicators to predict the efficacy of PD-1 inhibitors combination therapy in RM-NPC.

**Methods:**

The serum markers of 160 RM-NPC patients were measured before and three weeks after the first anti-PD-1 treatment. The least absolute shrinkage and selection operator (LASSO) logistic regression was carried out to select dynamic serum indicators and construct a prediction model. Furthermore, we carried out univariate, multivariate, nomogram and survival analyses to identify independent prognostic factors that were associated with 1-year progression-free survival (PFS).

**Results:**

Based on two markers that were screened by Lasso logistic regression, we constructed a risk score prediction model for the prediction of anti-PD-1 efficacy at 8–12 weeks with an AUC of 0.737 in the training cohort and 0.723 in the validation cohort. Risk score and metastases were included in the nomogram, and the Kaplan–Meier survival curves demonstrated that the high-risk group has shorter PFS compared to the low-risk group. The concordance index (C-index) of the nomogram for PFS is higher than that of the TNM stage in the training and validation cohort.

**Conclusion:**

We proposed a strategy to monitor dynamic changes in the biochemistry markers and emphasized their importance as potential prognostic biomarkers for the treatment of advanced NPC treated with PD-1 inhibitors. Our risk score prediction model was based on the dynamic change of LDH and AST/ALT, which has predictive and prognostic value for NPC patients who were treated with PD-1 inhibitors.

**Supplementary Information:**

The online version contains supplementary material available at 10.1186/s12935-021-02217-y.

## Introduction

Nasopharyngeal carcinoma (NPC) is one of the most common head and neck malignant tumors in Southeast Asia, including southern China [1, 2]. Concurrent radiotherapy and chemotherapy are the standard NPC treatments [2]. However, the available treatment options for patients with recurrence, distant metastasis, and resistance to first-line platinum-based chemotherapy are still very limited. Although the exact contribution of the Epstein-Barr virus (EBV) to the malignant transformation of epithelial tumors (i.e. undifferentiated NPC and EBV-related gastric cancer) remains unclear, the abnormal viral latent infection that was established by EBV in the epithelium is considered to be an important cause of malignant transformation. Further studies have also demonstrated that EBV infection can be identified in high-grade precancerous lesions of NPC, while it is rarely detected in low-grade precancerous lesions [3, 4].

Both the serum EBV antibody and plasma EBV DNA copy number are commonly used biomarkers for NPC diagnosis. With regards to early diagnosis and treatment of NPC, a large-scale prospective clinical study with more than 70,000 people was carried out in high-risk areas for NPC. By detecting two serum EBV antibodies, VCA-IgA and EA-IgA, and further examinations, the diagnosis rate was found to be significantly higher than that of the control group (79.0% vs. 45.9%, P < 0.0001) after six years of follow-up [5]. Another prospective study found that 78 patients (15.1%) out of 518 patients with non-metastatic NPC were plasma EBV DNA-negative (0–20 copies/ml), and 62 patients in this subset (12.0%) had 0 copy/ml [6]. Plasma EBV DNA was also used for efficacy judgment and prognostic analysis. However, it is usually used in primary treatment procedures. For patients that experience relapsed and refractory NPC, routine blood tests, blood biochemical tests, and imaging examinations are still most commonly used.

The immune-checkpoint-blockade (ICB) therapy, principally represented by PD-1/programmed death-ligand 1 (PD-L1) inhibitors, significantly improves the survival rate of patients with diverse cancer types. Encouraged by such great achievements, many clinical trials of ICB alone or with chemotherapy have been initiated in pre-treated patients with RM-NPC (recurrent or metastatic NPC) [7]. In clinical trials, certain therapeutic effects were observed, although the overall response rate was still unsatisfactory, and individual effects vary greatly [8–10]. Thus, there is an urgent need to find an effective prediction indicator for both treatment and survival benefits. A multicenter retrospective study reported that the combined baseline serum biomarkers are correlated with the efficacy of ICB treatments in NSCLC patients [11]. After evaluating 466 patients, the Lung Immune Prognostic Index (LIPI), the baseline derived neutrophils/(leukocytes minus neutrophils) ratio (dNLR) and LDH were identified as predictors of anti-PD-1 treatment in NSCLC. However, for RM-NPC patients who have received anti-PD-1 combined treatment, there is still a lack of reliable predictive serum markers for anti-PD-1 efficacy.

Herein, we conducted a retrospective study involving 160 NPC patients in order to explore the prognostic value of dynamic serum biomarkers. We aimed to explore reliable and minimally invasive prognostic indicators to predict the efficacy of anti-PD-1 combination therapy in NPC.

## Methods

### Patients and study design

From March 2018 to April 2021, this retrospective study included 160 patients from the Guangzhou Sun Yat-sen University Cancer Center who underwent the PD-1 checkpoint inhibitor combination treatment. The inclusion criteria included patients with recurrent or metastatic NPC who received PD-1 in combination with radiotherapy or chemotherapy from March 2018 to May 2020. Exclusion criteria included a follow-up time of < 1 year and a lack of hematological examination. Patients were randomly divided into two groups (ratio 2:1), including the training group (n = 106), which was used to construct the predictive model, and the validation group (n = 54), which helped validate the model. The PD-1 inhibitors used by patients included Camrelizumab, Pembrolizumab, Toripalimab, and Sintilimab, with dosages of 200 mg, 200 mg, 240 mg, and 200 mg, respectively, administered once every 3 weeks. PD-1 inhibitors were administered for at least three months, and complete blood counts and serum biomarkers were measured both at the beginning of treatment (within three days before the first treatment) and 3 weeks later. After treatment, the response was initially evaluated at 8–12 weeks and updated continuously. Demographic, clinical, and pathological data were also collected.

In the training and validation cohorts, radiological examinations were carried out according to RECIST (solid tumor response assessment criteria) v1.1 in order to evaluate the effect of immunotherapy at 8–12 weeks, which included complete response (CR), partial response (PR), stable disease (SD), and progressive disease (PD). The time interval between the start date of PD-1/PD-L1 inhibitor treatment, as well as the date of disease progression or death (PFS), was calculated for each patient.

The baseline covariates, which included age, body mass index (BMI), gender, clinical stage, histological type, Eastern Cooperative Oncology Group Performance Status (ECOG PS), metastasis stage, histological stages, clinical stages, and TNM classification were collected. Laboratory examinations, including white blood cell, counts, neutrophil counts, lymphocyte counts, monocytes, eosinophils, basophils, red blood cells, platelets, hemoglobin levels, neutrophil count/lymphocyte count (NLR), monocyte count/lymphocyte count(MLR), platelet count/lymphocyte count (PLR), carbon dioxide levels, calcium levels, lactate dehydrogenase (LDH) levels, creatinine levels, glucose levels, alanine aminotransferase (ALT) level, aspartate aminotransferase (AST) levels, bilirubin levels, gamma-glutamyl transpeptidase levels, alkaline phosphatase levels, cholinesterase (CHE) levels, creatine kinase levels, cystatin C levels, urea levels, uric acid (UA) levels, triglycerides, cholesterol levels, high-density lipoprotein-C, low-density lipoprotein-C, apolipoprotein A1 (ApoA1), apolipoprotein B, C-reaction protein, serum amyloid, total protein, globulin, and albumin levels. Details about how these serum biomarkers were measured are listed in Additional file 2: Table S1 and Additional file 3: Table S2.

### Statistical analysis

Disease control (DC) represents both partial response and stable disease. In order to construct the prediction model, we utilized the Lasso logistic regression in the training group to select markers. According to the regulation weight λ, LASSO shrinks all regression coefficients towards zero and sets the coefficients of many irrelevant features to zero. The optimal values of the penalty parameter λ were determined via tenfold cross-validation with the 1 standard error (SE) of the minimum criteria (the 1-SE criteria), whereas the final value of λ yielded a minimum cross-validation error. Retained features with nonzero coefficients were utilized for regression model fitting [12]. Next, the coefficients that were weighted by Lasso logistic regression were employed to calculate a risk score for each patient using a linear combination of selected variables.

The endpoint for the logistic regression was disease control at first scan after initiation of anti-PD-1 based therapy, which was between 8–12 weeks. The ROC curves, calibration curves, and clinical impact curves were utilized to determine the discrimination ability of the prediction model. Using Cox proportional hazard model, univariate and multivariate analyses were conducted to estimate the independent potential risk factors that affect 1-year PFS. The nomogram was established using data from the training cohort. Furthermore, the concordance index (C-index) and calibration curve were utilized to determine the predictive accuracy and discriminatory capacity. After establishing the nomogram, the optimal cut-off for the different continuous variables in the training cohort was determined by using the maximally selected rank statistics via the ‘surv_cutpoint’ function of the ‘survminer’ R package. Patients in the training and validation cohorts were then subdivided into high- and low-risk groups according to the optimal cutoff value, and Kaplan–Meier survival curves were then plotted for both groups. P < 0.05 was considered to be statistically significant. Analyses were performed with either the GraphPad Software or R Foundation for Statistical Computing.

## Results

### Patient characteristics and follow-up

The main clinical characteristics of the 160 NPC patients in the training and validation cohorts are listed in Table 1. Overall, 160 patients, including 113 males and 47 females were involved in this study. Patients ranged between 19 to 78 years old. The median age in this study was 47 years old in the training cohort, and 43 years old in the validation cohort. In our study, all patients were in the advanced TNM stage when using PD-1 inhibitors and underwent recurrence or/and lymph node metastasis or/and distant metastasis. Among the 160 patients, 73 experienced recurrence, while 87 did not have a recurrence. In addition, 83 patients had tumor distant metastasis while 77 did not have distant metastasis. The response to PD-1 inhibitor treatment in NPC patients was initially evaluated at 8–12 weeks and updated continuously. The follow-up time was more than 12 months. Patients were randomly subdivided into two groups at a ratio of 2:1. Results indicated that in the training and validation cohorts, 13.21% and 16.67% of patients, respectively, developed progressive disease, and 86.79% and 83.33% of patients respectively maintained disease control in the training and validation cohorts. Overall, 34 of 160 patients (21.25%) achieved a partial response.

**Table 1:** Demographics and clinical characteristics of patients

Characteristic cohort (n = 54)	Training cohort (n = 106) No. (%)	Validation No. (%)	p
Age, y			
Median (range)	47(24–73)	43(19–78)	0.9718
Sex			
Female	29 (27.36)	18 (33.33)	0.4660
Male	77 (72.64)	36 (66.67)	
Recurrence			
Yes	50 (47.17)	23 (42.59)	0.6180
No	56(52.83)	31 (57.41)	
ECOG			
0	6 (5.66)	9 (16.67)	0.1930
1	98 (92.45)	45 (83.33)	
2	2 (1.89)	0 (0.00)	
Histological type			
Poorly differentiated type	3 (2.83)	6 (11.11)	0.0620
Undifferentiated type	103 (97.17)	48 (88.89)	
Clinical stage			
II–III	38 (35.85)	19 (35.19)	0.1430
IVa	21 (19.81)	15 (27.78)	
IVb	36(33.96)	12 (22.22)	
IVc	11 (10.38)	8 (14.81)	
Tumor stage			
T0–2	24 (22.64)	12 (22.22)	0.9520
T3–4	82 (77.36)	42 (77.78)	
Node stage			
N0–1	47 (44.34)	23 (42.59)	0.8670
N2–3	59 (55.66)	31 (57.41)	
Metastasis stage			
M0	49 (46.23)	28 (51.85)	0.5090
M1	57 (53.77)	26(48.15)	
Outcomes			
PR	24 (22.64)	10 (18.52)	0.0610
SD	68 (64.15)	35 (64.81)	
PD	14 (13.21)	9 (16.67)	
The PD1 drug			
Camrelizumab	16 (15.09)	2 (3.70)	0.2070
Pembrolizumab	3 (2.83)	0 (0.00)	
Toripalimab	72 (67.93)	45 (83.33)	
Sintilimab	15 (14.15)	7 (12.97)	

*ECOG* Eastern Cooperative Oncology Group Performance Status, *PR* partial response, *SD* stable disease; *PD* progressive disease

### Construction of the risk model based on the dynamic changes of serum markers to predict clinical outcomes

**Fig. 1:**
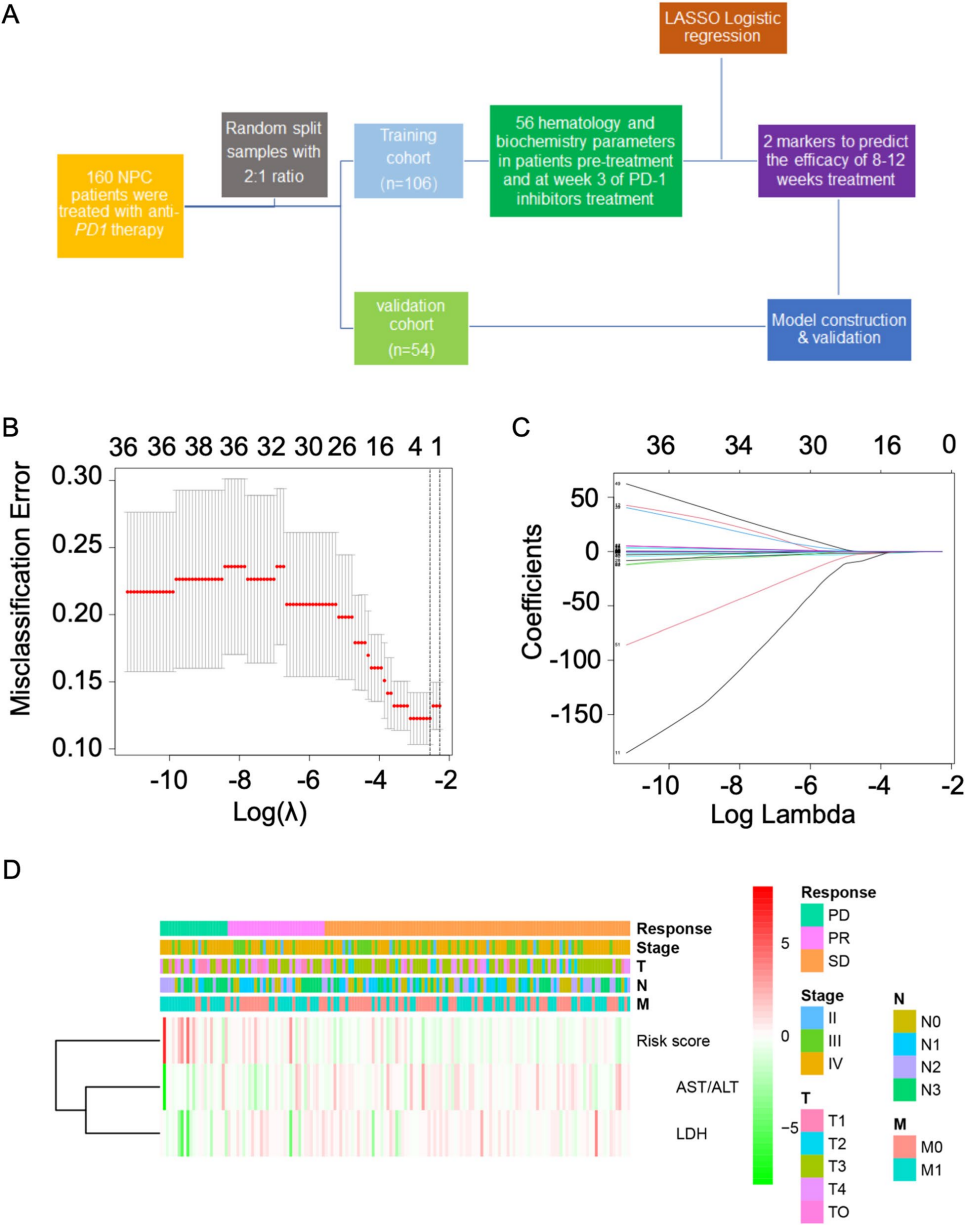
Prediction of the efficacy of NPC patients treated with PD-1 inhibitors based on dynamic serum indicators. **A** Workflow for modeling and validation of prediction model; **B** LASSO regression analysis for candidates screening; **C** Coefficients of selected markers; **D** Unsupervised hierarchical clustering of selected markers and clinical outcomes

LASSO regression is suitable for analyzing high-dimensional data as it is able to extract the most important predictor variables from the original data set. The differences between the 56 serum markers of NPC patients after three weeks of PD-1 inhibitors treatment and before treatment were calculated (∆_W3_). LASSO logistic regression model was applied in order to build a risk score prediction model and 56 variables were then reduced to two potential predictors. A coefficient profile plot is shown in Fig. 1B, and a cross-validated error plot is shown in Fig. 1C. Our model demonstrated that two dynamic serum indicators (dynamic changes of AST/ALT, and LDH) that were identified by the training set (n = 106) were utilized to predict the efficacy of anti-PD-1 therapy (Fig. 1D).

The risk score of each patient was obtained using regression coefficients of dynamic changes of markers: Risk score = (− 0.242*∆_W3_ AST/ALT) + (− 0.0007*∆_W3_LDH).

### The performance of the prediction model

**Fig. 2:**
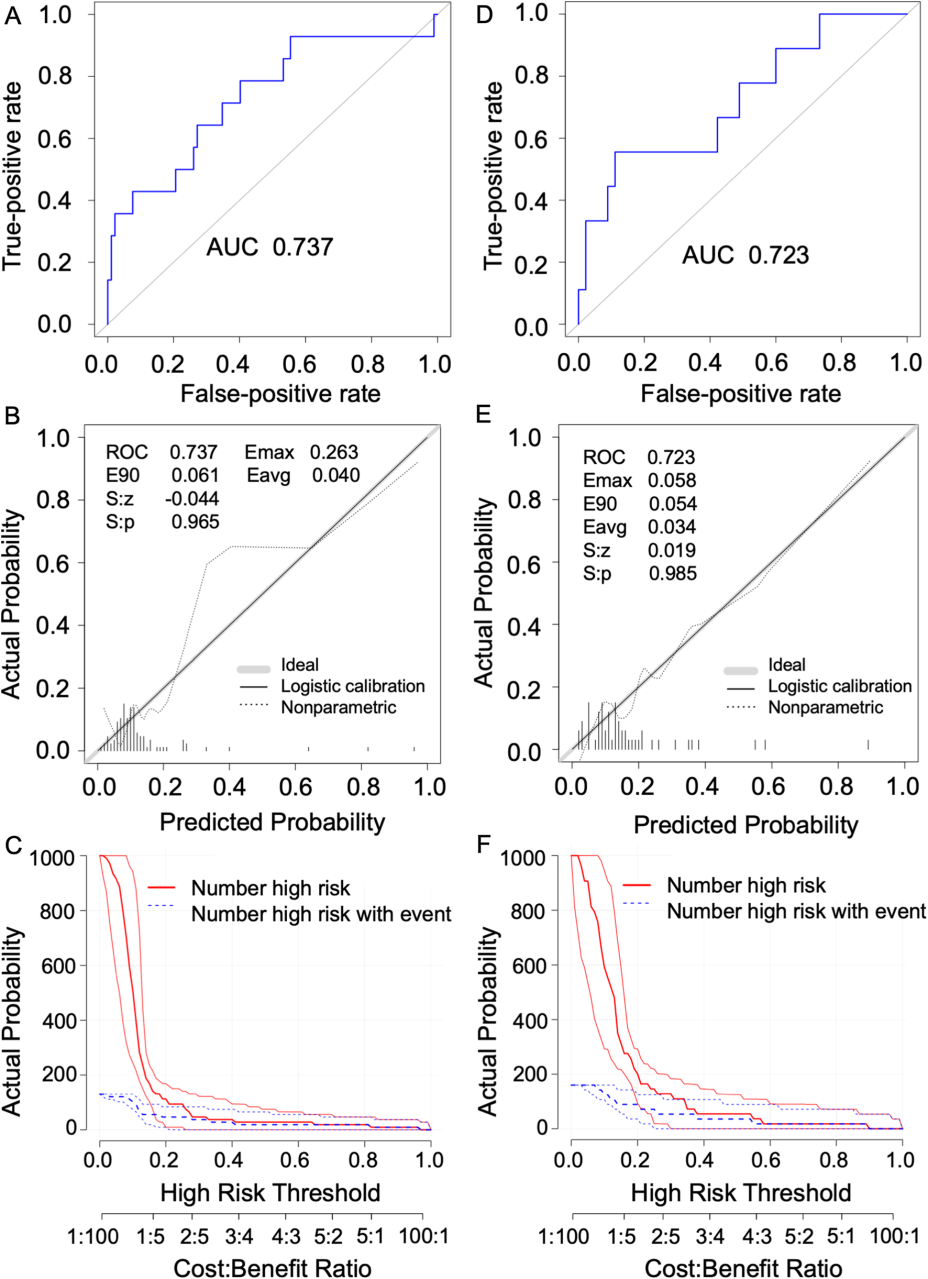
The prediction accuracy of the efficacy prediction model for NPC patients treated with PD-1 inhibitors. **A** Receiver operating characteristic (ROC) curves of a prediction model in the training cohort; **B** Calibration curve of a prediction model in the training cohort; **C** Clinical impact curve of a prediction model in the training cohort; **D** Receiver operating characteristic (ROC) curves of a prediction model in the validation cohort; **E** Calibration curve of a prediction model in the validation cohort; **F** Clinical impact curve of a prediction model in the validation cohort

The risk score obtained by our model demonstrated superior sensitivity and specificity for anti-PD-1 efficacy prediction [area under curve (AUC), 0.737; Fig. 2A] within the training cohort. Our model demonstrated the consistency of the probability of efficacy prediction between the optimal prediction, as well as actual observation (Fig. 2B). Besides, the decision curve analysis (DCA) also suggested the potential clinical effects and its utility of risk score (Fig. 2C).

Afterward, we validated our model within the validation cohort. The ROC analysis demonstrated slightly lower sensitivity and specificity than the training cohort [area under curve (AUC), 0.723; Fig. 2D]. The ROC analysis demonstrated the agreement of the probability of efficacy prediction between the optimal prediction and actual observation within the validation cohort (Fig. 2E). Besides, the decision curve analysis (DCA) also suggested the potential clinical effects and its utility of risk score as a similar clinical impact curve was obtained in the validation cohort (Fig. 2F).

### Risk score and metastasis stage associated with 1-year PFS

**Fig. 3:**
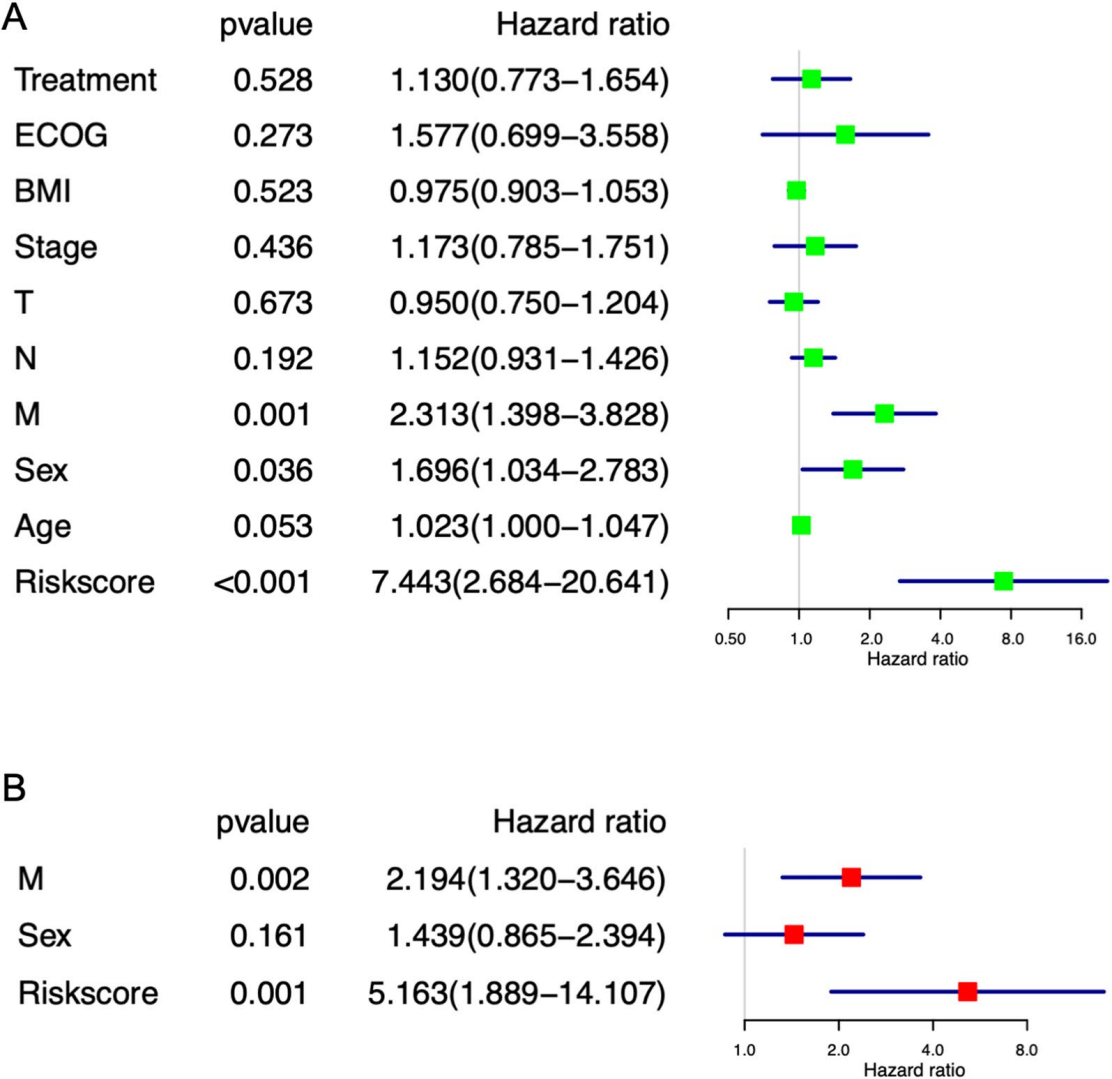
Univariate and multivariate Cox regression analyses. **A** Univariate analysis of clinical characteristics and risk score in the combined cohort; **B** Multivariate analysis of selected variances

We performed a univariate analysis that included nine covariates, including treatment pattern, ECOG, BMI, clinical stage, TNM classification, gender, and age. Metastasis stage, gender and risk score were significantly associated with 1-year PFS [Hazard ratio (HR) = 2.313, 95% CI 1.398–3.828, p = 0.001; HR = 1.696, 95% CI 1.034–2.783, p = 0.036; and HR = 7.443, 95% CI 2.684–20.641; p < 0.001, respectively] (Fig. 3A). There were no differences observed in our cohorts according to treatment pattern, ECOG, BMI, clinical stage, tumor stage, node stage, and age with regards to 1-year PFS. In multivariate analysis (Fig. 3B), metastasis stage and risk score were found to be strongly associated with 1-year PFS [Hazard ratio (HR) = 2.194, 95% CI 1.320–3.646, p = 0.002; and HR = 5.163, 95% CI 1.889–14.107, p = 0.001, respectively]. Gender appeared to have little effect on 1-year PFS, and there is no significant difference in the multivariate analysis. Meanwhile, considering the significant difference in the incidence rate of NPC between males and females, we did not include gender within the candidate range of predictors.

### Nomogram development with risk score and metastasis stage

**Fig. 4:**
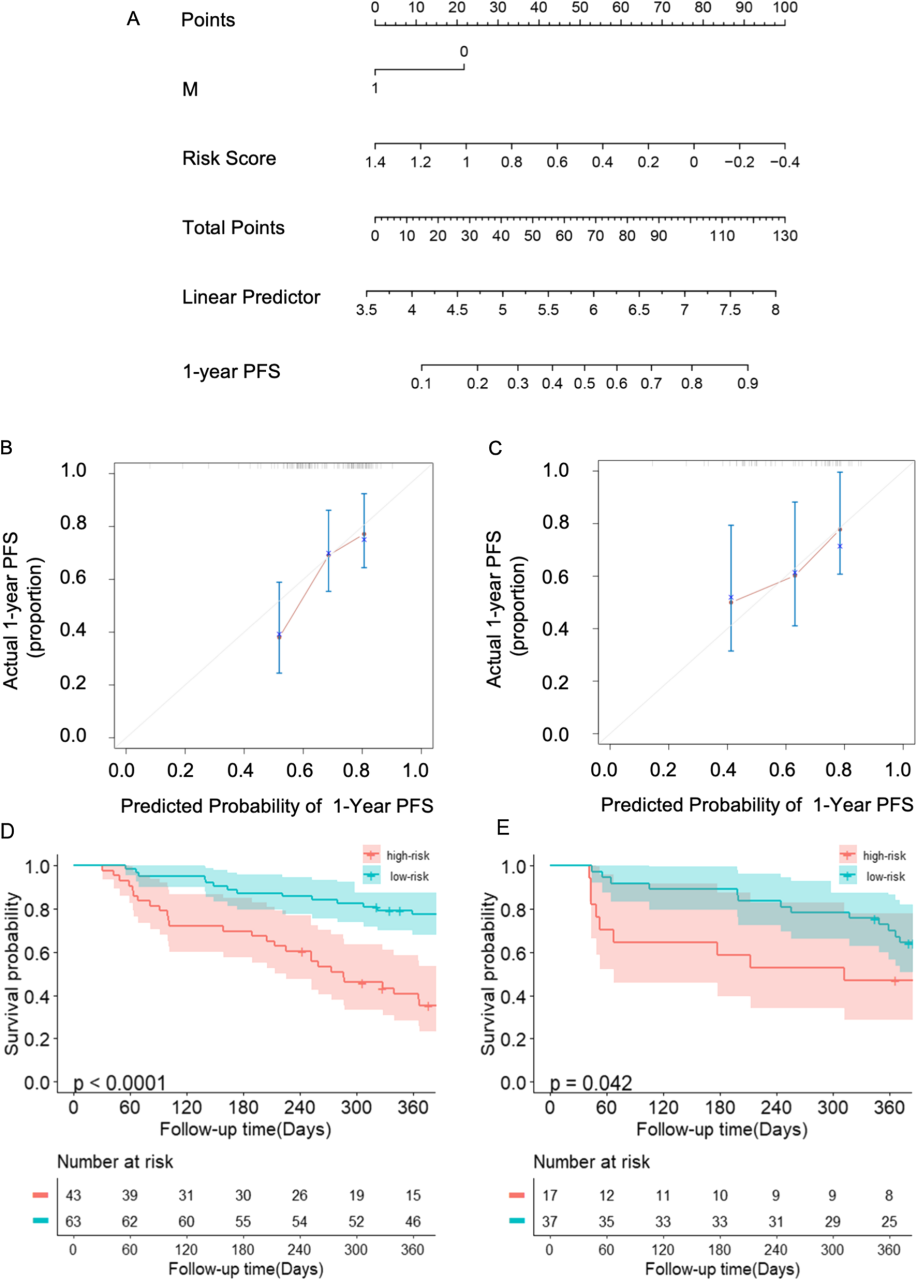
Prognostic prediction 1-year PFS of NPC patients treated with PD-1 inhibitors based on risk scores and metastasis stage. **A** Nomogram predicting the 1-year PFS in the training cohort; **B**, **C** the calibration plot for the nomograms at 1- year PFS in training cohort (**B**) and validation cohort (**C**); **D**, **F**. Kaplan–Meier curves for 1–year PFS based on the predictions of the nomogram in the training cohort (**E**) and the validation cohort (**F**)

We constructed a nomogram using results from multivariate analysis (Fig. 4A). We included risk score and metastasis into the nomogram. Selection of the final cutoff point was conducted using the R package “survival”. The calibration curve of the nomogram is shown in Fig. 4B and C, which was derived from the training cohort and the validation cohort, respectively.

Subsequently, the patients were either subdivided into the high-risk or low-risk group according to “Auto Select best cutoff” (Additional file 1: Fig. S1) using the Kaplan–Meier plotter. High risk indicated that a total score of ≤ 78.5, while the low risk indicated a total score of > 78.5. The Kaplan–Meier survival curves demonstrated that the high-risk group has a shorter PFS compared to the low-risk group [(p < 0.001, the training cohort; p = 0.042, the validation cohort), Fig. 4D–E]. The concordance index (C-index) of the nomogram for PFS was 0.662 (95% CI 0.577–0.746), which was higher than that of the TNM stage [0.536 (95% CI 0.465–0.606)] in the training cohort (Table 2). In the validation cohort, the C-index of the nomogram for predicting PFS was also higher compared to that of the TNM stage [0.630 (95% CI 0.509–0.752) vs. (0.513; 95% CI 0.410–0.615)].

**Table 2:** C-index for the prediction of PFS in the study cohorts

Factors	Training cohort		Validation cohort		Total cohort	
	C-index (95% CI)	P-value	C-index (95% CI)	P-value	C-index (95% CI)	P-value
TMN stage	0.53 (0.465–0.606)		0.513 (0.410–0.615)		0.518 (0.460–0.577)	
Nomogram	0.662 (0.577–0.746)		0.630 (0.509–0.752)		0.650 (0.581–0.718)	
Nomogram vs TNM stage		0.002		0.105		< 0.001

*C-index* concordance index, *CI* confidence interval, *TNM* tumor/node/metastasis

Nomogram: Risk scores + M stage

P-values are calculated based on normal approximation using rcorrp.cens package function in Hmisc in R

## Discussion

Increasingly more patients with advanced-stage cancer benefit from PD-1/PD-L1 inhibitors. Despite the certain success in clinical trials of PD-1 inhibitors for NPC, not all patients are able to achieve long-term improvement in progression-free survival.

Thus far, scientists have developed many predictive markers for PD-1 inhibitor efficacy [13], including PD-L1 expression [14], tumor mutational burden (TMB) detection [15], microsatellite instability (MSI) detection [16], dMMR (mismatch repair) detection, tumor-Infiltrating lymphocyte (TIL) detection [17], and even intestinal flora analysis [18].

Despite the fact that there is evidence that these above biomarkers are valuable for predicting the effect of immunotherapy, these tests are generally expensive, with low penetration rates, as well as large regional differences in detection rates. The cost of TMB testing involves next-generation sequencing, which is even more prohibitive [19]. In addition, for tumor tissue PD-L1 staining, it was difficult to standardize and unify the pathological section staining and image reading among the different medical institutions, which may cause the reliability of the results to be greatly reduced. For patients with RM-NPC whose primary tumor has been cleared, biopsy for PD-L1 staining is inappropriate when undergoing PD-1 combined with radiotherapy or chemotherapy as the second-line treatment. In contrast, blood counts and serum chemical composition testing are easy to achieve, and cancer patients regularly undergo testing during treatment. It is worth mentioning that blood markers related to systemic inflammation and tumor burden are known to be related to immunotherapy response.

Lactate dehydrogenase (LDH) is a classic inflammatory marker found in cancer patients [20]. Lactate is not only a type of waste product in cellular metabolism but also functions as a critical molecule involved in cancer. As previously reported, lactate connects cancer cells, immune cells, and stromal cells in the tumor microenvironment [21]. LDH provides energy for tumor cells. Under hypoxic conditions, the growth of LDH-deficient tumor cells becomes limited [22]. It has been shown that tumor cells with decreased LDH activity are not able to maintain high ATP levels, which results in slow cell proliferation under normoxic or hypoxic conditions. The most common mechanism of LDH regulating cell migration and invasion is known to be related to lactate secretion. Studies have shown that the level of lactic acid is related to the incidences of distant metastasis, and a high concentration of lactic acid is related to the early distant metastasis rate of cancer [23].

More importantly, LDH can be utilized as a prognostic index of malignant tumor [24]. LDH is one of the risk factors in the international prognosis index (IPI), and a strong predictor of survival among patients with invasive lymphoma [25]. Studies have also reported the correlation between LDH serum levels and clinical outcomes in Ewing's sarcoma [26], in advanced biliary tract cancer [27], renal cell carcinoma [28] with chemotherapy, and in melanoma [29, 30], advanced non-small cell lung cancer [31] with PD-1 treatment.

The levels of serum ALT/AST ratio (LSR) have been generally accepted as a predictor of liver injury [32]. One study reported that baseline ALT/AST is related to the prognosis of patients with gastric adenocarcinoma [33]. In addition, GGT and AST/ALT are independent factors that predict the overall survival rate of esophageal squamous cell carcinoma [34] and primary hepatic carcinoma [35].

Herein, we demonstrated that the dynamic changes of LDH and LSR are related to the efficacy of PD-1 inhibitors in NPC, as well as the prognosis of RM-NPC patients. Meanwhile, as the dosing interval, three weeks after the initiation of treatment may be an appropriate time to evaluate the correlation between biomarkers, and an anti-PD-1 antibody treatment response in NPC patients, according to previous research [36]. As one of the indicators we selected, LDH has been validated to have prognostic value in chemotherapy and immunotherapy while ALT/AST needs to be confirmed in more studies. Thus, our study may provide clues for the use of these blood parameters as biomarkers for checkpoint inhibitors. As an important part of our study, we utilized the Lasso analysis to explore potential serum markers that established and verified our predictive prognostic model. Compared to the most adopted PD-L1 expression or TMB detection, monitoring the dynamic changes of serum markers was less invasive and more economical. Through the use of the predictive model, we were able to predict the short-term efficacy of 8–12 weeks and 1-year PFS according to dynamic changes of serum markers before and three weeks after the first anti-PD-1 treatment. One strength of this study is the nomogram combined by risk score and metastasis stage in predicting the prognosis of patients with RM-NPC, and the high-risk group had a shorter 1-year PFS than the low-risk group.

To the best of our knowledge, our study is the first to explore prognostic parameters in RM-NPC patients who were treated with anti-PD-1 inhibitors. A large-scale cohort study indicated TILs may reflect the immunological heterogeneity of NPC and may represent a new prognostic biomarker [37]. However, this study is not just for patients who underwent anti-PD-1 therapy. The same group also established an immune score model that estimates the risk of disease progression in NPC patients [38]. Nevertheless, our model focuses on the dynamic changes of existing serum parameters in NPC patients, avoids additional costs, and provides a novel predictive model for patients who have undergone expensive anti-PD-1 therapy.

Considering the nature of retrospective studies, our study still has some limitations, which include insufficient sample size due to missing laboratory data, lack of standardized predictive biomarkers as a reference, and the diversity of pre-treatment models. In addition, our observational research was based on a single institution, which may cause selection bias.

## Conclusions

In summary, we proposed a strategy to monitor dynamic changes of biochemistry markers and emphasized their importance as potential prognostic biomarkers for the treatment of advanced NPC with ICBs. As our model may help identify RM-NPC patients who are unlikely to benefit from anti-PD-1 therapy, further investigations are needed in order to evaluate the predictive value of these markers in larger multicenter populations and prospective clinical studies.

## Supplementary Information

**Additional file 1:Figure S1.** The optimal cut-off value of prognostic model using the R package “survival”.

**Additional file 2:Table S1.** Test items, test methods, and instrument of serum markers.

**Additional file 3:Table S2.** Abbreviations and full names of serum markers.

## Data Availability

Primary datasets used and/or analyzed during the study have been generated and deposited in the RDD system (http://www.researchdata.org.cn) with the approval RDD number as RDDA-2021724080.
