# Correction to: Dynamic serum biomarkers to predict the efficacy of PD-1 in patients with nasopharyngeal carcinoma

**DOI:** 10.1186/s12935-021-02369-x

**Published:** 2021-12-02

**Authors:** Ao Zhang, Guanqing Zhong, Luocan Wang, Rongzeng Cai, Runkun Han, Caixia Xu, Shulin Chen, Peng Sun, Hao Chen

**Affiliations:** 1 Department of Clinical Laboratory, State Key Laboratory of Oncology in South China, Collaborative Innovation Center for Cancer Medicine, Guangdong Key Laboratory of Nasopharyngeal Carcinoma Diagnosis and Therapy, Sun Yat-Sen University Cancer Center, Guangzhou, 510060 People’s Republic of China; 2 Research Center for Translational Medicine, The First Affliated Hospital, Sun Yat-Sen University, 58 Zhongshan Road 2, Guangzhou, 510080 Guangdong People’s Republic of China; 3 Department of Medical Oncology, State Key Laboratory of Oncology in South China, Collaborative Innovation Center for Cancer Medicine, Guangdong Key Laboratory of Nasopharyngeal Carcinoma Diagnosis and Therapy, Sun Yat-Sen University Cancer Center, Guangzhou, 510060 People’s Republic of China

## Correction to: Cancer Cell Int (2021) 21:518 https://doi.org/10.1186/s12935-021-02217-y

In this article [1], the affiliation details for Shulin Chen and Peng Sun were incorrectly given as “1” and “2,3” but should have been “1,2” and “3”, respectively.

The affiliations are corrected with this correction. The correct unit labeling should be as follows:

Ao Zhang^1^, Guanqing Zhong^1^, Luocan Wang^1^, Rongzeng Cai^1^, Runkun Han^1^, Caixia Xu^2^, Shulin Chen^1,2*^, Peng Sun^3*^ and Hao Chen^1*^
