# Supplementary figures and images for: Dynamic serum biomarkers to predict the efficacy of PD-1 in patients with nasopharyngeal carcinoma
Source: Cancer Cell Int. 2021 Sep 28;21:518. doi: 10.1186/s12935-021-02217-y (PMC8480072; doi:10.1186/s12935-021-02217-y)

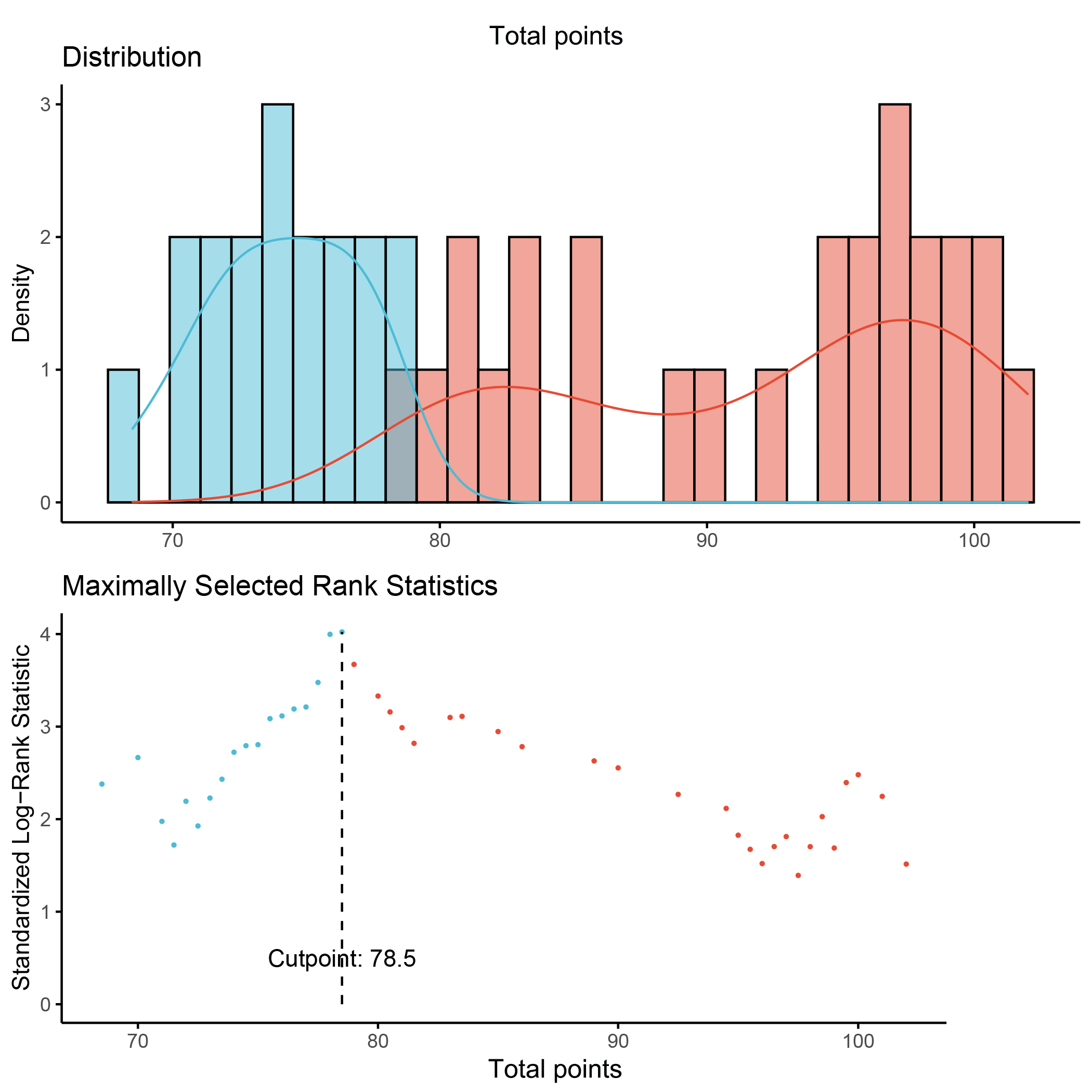

Supplement: Supplementary file 1 — Additional file 1:Figure S1. The optimal cut-off value of prognostic model using the R package “survival”. [file 12935_2021_2217_MOESM1_ESM.tif]
