# Supplementary material for: Dynamic serum biomarkers to predict the efficacy of PD-1 in patients with nasopharyngeal carcinoma
Source: Cancer Cell Int. 2021 Sep 28;21:518. doi: 10.1186/s12935-021-02217-y (PMC8480072; doi:10.1186/s12935-021-02217-y)
Supplement: Supplementary file 3 — Additional file 3:Table S2. Abbreviations and full names of serum markers. [file 12935_2021_2217_MOESM3_ESM.docx]

Supplementary Table.S2

| Abbreviation | Full name |
| --- | --- |
| Ca2+  CO2  CK  CHE  BUN  SAA  CRP  ALT  CYSC  HDL  LDL  TBIL  DBIL  GGT  TP  APOA1  APOB  AST  ALP  LDH  TBA  TG  CHO  GLU  UA  CRE  ALB  WBC  RBC  PLT  HGB  SII  GLB  HCT  MCV  MCH  MCHC  RDW-CV  RDW-SD | calcium  carbon dioxide  creatine kinase  cholinesterase  urea  serum amyloid protein  C-reactive protein  alanine aminotransferase  cystatin C  high density lipoprotein  low density lipoprotein  total bilirubin  direct bilirubin  glutamyltransferase  total protein  apolipoprotein A1  apolipoprotein B  aspartate aminotransferase  alkaline phosphatase  lactate dehydrogenase  total bile acid  triglyceride  cholesterol  glucose  uric acid  creatinine  albumin  white blood cell  red blood cell  platelet  hemoglobin  systemic immune-inflammation index  globulin  hematocrit  mean erythrocyte volume  mean erythrocyte hemoglobin  mean erythrocyte hemoglobin concentration  coefficient of variation of erythrocyte distribution width  standard deviation of erythrocyte distribution width |
